# Investigation of the Occurrence of Zoonotic Intestinal Parasites along the Karmanasa River Bank in Lalitpur, Nepal

**DOI:** 10.1002/vms3.70164

**Published:** 2024-12-18

**Authors:** Roshan Babu Adhikari, Diksha Ghimire, Tirth Raj Ghimire

**Affiliations:** ^1^ Department of Zoology Nepalese Army Institute of Health Sciences (NAIHS) Kathmandu Nepal; ^2^ Department of Zoology Alka Health Institute Pvt. Ltd. Lalitpur Nepal; ^3^ Wildlife Biology Third Pole Conservancy (TPC) Bhaktapur Nepal; ^4^ Department of General Medicine Alka Health Institute Pvt. Ltd. Lalitpur Nepal; ^5^ Department of Zoology Tri‐Chandra Multiple Campus, Tribhuvan University Kathmandu Nepal

**Keywords:** *Cryptosporidium*, *Entamoeba*, GI parasites, hookworm, rats, zoonosis

## Abstract

**Introduction:**

Increasing urbanization has particularly affected rivers and their outer edges in cities, including Kathmandu Valley, which encompasses Lalitpur, the nation's third‐largest city. This study aims to conduct a parasitological survey to investigate the occurrence of zoonotic intestinal protozoa and helminths along the Karmanasa River bank in central Nepal.

**Methods:**

Faecal samples from openly defaecating animals were collected via non‐invasive techniques, and coproscopy was carried out using direct wet mount, concentration and acid‐fast staining methods to ensure reliable findings.

**Results:**

The findings showed that all the faecal samples were positive for intestinal parasites as follows: buffaloes (15/15), cats (5/5), cattle (30/30), chickens (7/7), dogs (15/15), goats (15/15), pigs (20/20) and rats (12/12). A total of 28 intestinal parasites were reported, out of which 21 species possess zoonotic potentialities, and each host was reported to harbour at least one zoonotic parasite. *Entamoeba* sp., *Cryptosporidium* sp., hookworm, *Trichuris* sp., *Trichostrongylus* and *Balantidium coli* were among the predominant zoonotic parasites. The use of the landscape for livestock grazing and the presence of free‐ranging animals could have all added to the zoonotic risks. Moreover, the excretion of a moderate to large number of zoonotic parasite eggs in the faecal samples indicates a transmission risk.

**Conclusions:**

The study detected 21 species of intestinal zoonotic parasites circulating along the landscape in the study area, indicating a higher risk of cross‐transmission. Therefore, strategic treatment of livestock and free‐ranging animals and periodic public health awareness programs for the local inhabitants are highly recommended.

## Graphical abstract

Summary

• Twenty‐one intestinal zoonotic parasites out of 28 were detected.

• Parasitic burden measured by eggs per gram (EPG) was heavy on the river bank.

• Cross‐transmission of intestinal parasites among co‐habiting animals may occur.

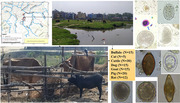

## 1 Introduction

Gastrointestinal parasites (GIPs) comprise single‐celled protozoa and multicellular helminths, one of the major pathogens of clinical and veterinary significance (Góralska and Błaszkowska 2015; Haque 2007). These parasites are common in developing countries such as Nepal because of their higher prevalence rate, ease of transmission and, more importantly, the diagnostic and treatment complexities (R. B. Adhikari, Parajuli, et al. 2021; Devleesschauwer et al. 2014). Notably, they are the etiological agents of gastrointestinal illness in humans and animals and are directly associated with substantial morbidity, mortality and economic burdens (Ghimire, Regmi, and Huettmann 2020). Although most GIPs are specific to their natural host (O'Donoghue and Pryor 2010), some can infect other hosts, including humans in multi‐host–pathogen systems, known as zoonotic parasites (Bowden and Drake 2013). The proximity and intimate association of humans with co‐habiting hosts such as domestic animals, pets and wildlife in a typical landscape may result in the pathogen cross‐transmission phenomenon in nature (Barnes et al. 2017; Thompson and Kutz 2019). However, food and behavioural habits, a lack of clean drinking water, environmental pollution and the availability of potential vectors may contribute to the associated risk (Barnes et al. 2017; Barton et al. 2023; Macpherson 2005). Notably, humans are not the definitive host of zoonotic parasites, so these parasites act differently in humans (Blackwell 2015). However, they can induce severe pathological effects, including growth retardation, malnutrition, anaemia, diarrhoea, neurological disorders, reproductive as well as congenital distress and even death. This is why zoonotic GIPs are a severe public health concern with global distribution (Barnes et al. 2017). The main zoonotic GIPs include *Entamoeba*, *Giardia*, *Cryptosporidium*, *Toxoplasma*, *Trichinella*, *Fasciola* and various tapeworm, roundworm, hookworm and whipworm species. These parasites are either food‐borne, water‐borne or soil‐borne and can infect humans alone or in combination with animals owing to their monogenetic or digenetic lifecycle (Slifko, Smith, and Rose 2000).

Due to diverse public health issues and the potential to spread diseases and pandemics, the study of zoonotic parasites has proven to be an exciting area of research with greater relevance in developing countries such as Nepal. Concerning it, a systematic review reported that 20 different zoonotic diseases, such as intestinal protozoa and helminths, neurocysticercosis, congenital toxoplasmosis, cystic echinococcosis, trichinellosis, toxocariasis and schistosomiasis, imposed a significant public health burden in Nepal (Devleesschauwer et al. 2014). Another review examining the risk of common zoonosis, including taeniasis and cysticercosis, among livestock farmers concluded that Nepalese farmers needed proper knowledge and management measures to prevent such diseases (R. Adhikari and Bagale 2019). In these circumstances, Nepal remains at risk for parasitic zoonosis caused by GIPs. Furthermore, Nepal often faces constraints in healthcare infrastructure, making it challenging to identify and control disease outbreaks quickly. Hence, periodic monitoring of zoonotic pathogens across various agricultural regions is essential to prevent widespread transmission.

Nonetheless, this critical aspect of health threats lacks attention and has not been prioritized by government bodies, public health workers and veterinarians in Nepal. In addition, the transmission route and the potential impact are only evaluated if humans in the shared environment are infected with any zoonotic parasitic species found in these co‐habiting animals. Therefore, using copro microscopic techniques, the current study aims to investigate the occurrence of intestinal zoonotic parasites in the faecal samples of openly defaecating livestock and free‐ranging animals co‐habiting along the Karmanasa River bank in central Nepal.

## 2 Methodology

### 2.1 Study Area

**FIGURE 1:**
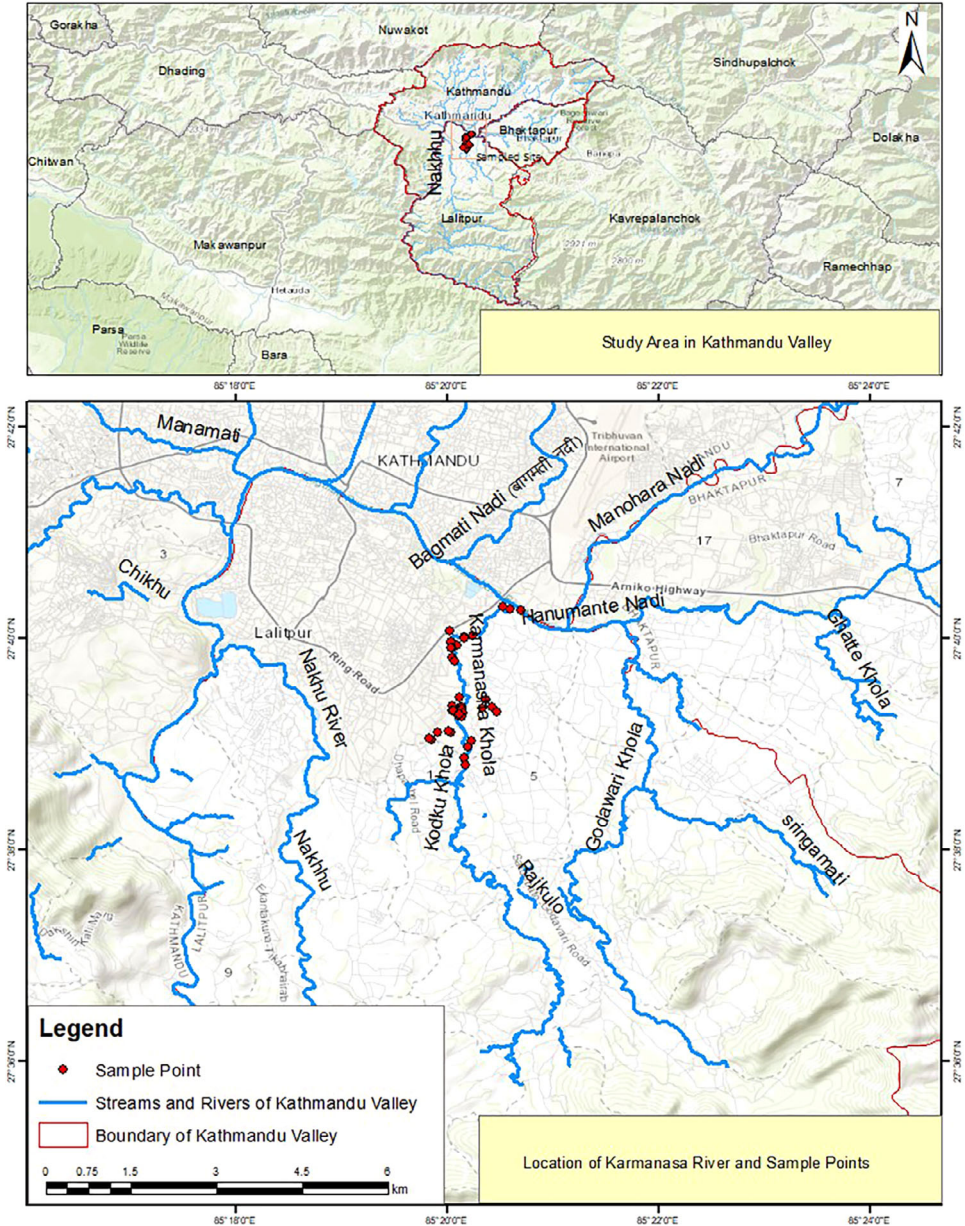
Map of the study area.

**FIGURE 2:**
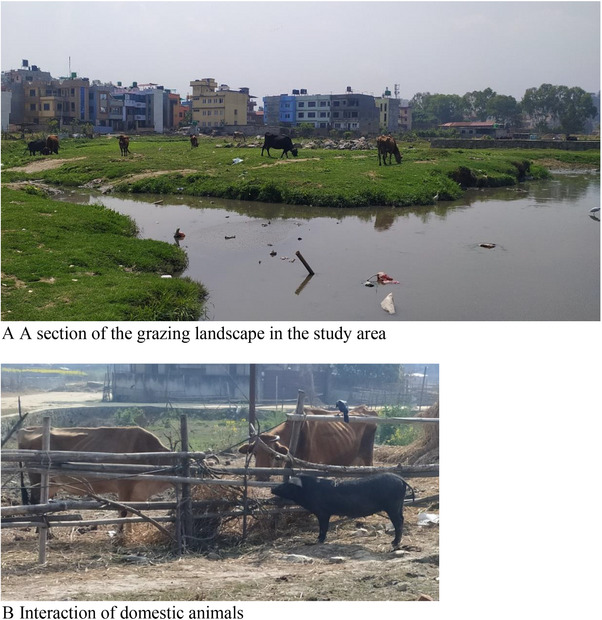
Landscape of the study area. (A) Grazing landscape (B) Interaction of domestic animals

The study has been conducted in the Kodku Khola River basin, also called Karma Nasa (27°39′20.6″ N, 85°20′08.2″ E) (Figure 1). It originates at Guindaha of Badikhel village panchayat, which is currently a part of Godawari Municipality in the Lalitpur District (Shrestha 1988). It is the sole river system that flows from south to north direction. It passes through Godawari, Hattiban and Gwarko and ends at the Balkumari Manohara River in Kathmandu. Nonetheless, the surrounding regions from Hattiban to the Manohara River are included in our study area. Lalitpur experiences humid subtropical weather. Even though the region receives reasonably distributed rainfall throughout the year, ranging from 153 to 241 mm, the temperature is relatively high, with the highest yearly range being 18–29°C and the lowest range being 3–20°C (R. B. Adhikari, Adhikari Dhakal, and Ghimire 2023). The area also includes open landscapes for livestock grazing and for canine and felid free‐roaming, as well as for playing and recreational activities, such as morning walks and yoga for humans (Figure 2).

### 2.2 Faecal Sample Collection, Preservation and Transportation

The field and laboratory studies were conducted from November 2021 to December 2023. The nearby areas of the Karmanasa River were visited, and opportunistically, 117 faecal samples, one from each participating animal belonging to eight different species, were collected. These animals included livestock, free‐ranging cats and dogs and rodents that defaecate in the open landscape of the study area. In addition, verbal consent was obtained from the livestock owners, and non‐invasive sampling techniques were used to collect faecal samples. The faecal samples that fell immediately after defaecation were only considered and collected. To prevent the duplication of samples, the grazing animals such as cattle, buffaloes, goats and pigs, including the free‐ranging animals, were tracked and followed until they defaecated in the open landscape. In addition, photos of each animal were obtained for visual reference. After defaecation, a wooden applicator and gloved hands were used to pick up the uncontaminated portion of the faecal samples to put them into 20 mL sterile vials.

In addition, we used artificial sand trays or boxes to collect the faecal samples of domestic felines based on the protocol previously explained (R. B. Adhikari, Adhikari Dhakal, et al. 2023). We placed four wooden sand trays filled with sand at different preferable sites and put bread and homemade flatbread to attract them. Later, the cats visited these trays, took the food, accepted the trays and defaecated there.

Similarly, the habitat trap method was applied to collect the droppings of rats. Three metallic cages were used with bread and homemade flatbread as bait near the preferred roosting sites to trap them. The trapped rats were then allowed to eat all the baits. We waited till they defaecated and then finally released without harming them.

All the collected faecal samples were examined macroscopically for the presence or absence of blood, mucus and worm segments, which ensured the accuracy of the findings. Then, 2.5% (w/v) potassium dichromate solution (K_2_Cr_2_O_7_) was added to each vial. The vials were then carried to the laboratory for microscopic analysis.

### 2.3 Laboratory Processing and Examination

The copromicroscopic techniques used in this study involved four specific laboratory techniques as follows: direct wet mount, formal‐ether sedimentation, zinc sulphate flotation and acid‐fast staining methods. These techniques followed the protocol previously explained in the literature (R. B. Adhikari, Adhikari Dhakal, and Ghimire 2024; R. B. Adhikari, Ale, et al. 2022; Ghimire, Adhikari, and Bhattarai 2021).

First, faecal pellets were crushed using a glass rod and stirred gently in the preservative media. For the direct wet mount technique, a single drop of this sample was spread over the sterile glass slide and examined under the microscope (40×) by covering it with a coverslip. A slightly higher volume of samples (about 2 g) was taken for the formal‐ether sedimentation technique. The faecal suspension was centrifuged (at 2000 rpm for 3 min) with 12 mL of normal saline and then with a mixture of 10 mL of 10% formalin and 4 mL of ethyl acetate. Finally, after discarding the supernatant, a single drop of sediment was spread over the glass slide and observed under the microscope (40×).

Similarly, for the flotation technique, the sediments obtained after initial centrifugation were added with 32% (w/v) of zinc sulphate (ZnSO_4_) solution and centrifuged (at 2000 rpm for 3 min). Additional flotation media were added gently to fill the centrifuge tube without discarding the supernatant, and a coverslip was placed on the top. After 5–10 min, the coverslip was removed and observed under the microscope (40×).

For the acid‐fast staining technique, the sediments obtained after formal‐ether sedimentation were used for thin smear preparation. These smears were dried at room temperature and fixed in absolute methanol for 2 min. The smear was further flooded with carbon fuchsin, gently heated for about 5 min, then washed with acid alcohol and distilled water. The smear was then restained with malachite green and rinsed gently with cool distilled water. Finally, after air drying, the smear was observed under the microscope (100×) using immersion oil.

### 2.4 Estimation of Parasitic Burden/Severity of Infection

We used the ‘A 2 Cell McMaster Counting Slide’ (Hawksley and Sons Ltd.) to accurately determine the parasitic infection level. Our methodology involved calculating the number of eggs of helminths released in every gram of faecal matter to establish the infection level. To ensure precision and consistency, the manufacturer's instructions were followed, as previously explained in the literature (R. B. Adhikari, Adhikari Dhakal, et al. 2021).

### 2.5 Parasite Identification

All the morphological stages of parasites, including cysts, oocysts and trophozoites of protozoa and eggs and larvae of helminths, were imaged under a microscope using a mobile camera. Parasite identification depended entirely on morphometric analysis, as explained in previously published research (Soulsby 2012; Zajac and Conboy 2012). The identification of *Fasciola* sp. was confirmed with a golden yellow colour with methylene blue, while *Paramphistomum* sp. remained colourless (R. B. Adhikari, Adhikari Dhakal, and Ghimire 2022).

### 2.6 Data Analysis

All the collected and generated data were organized in tables using Microsoft Word 2016 and Microsoft Excel 2016. To calculate the percentage prevalence rate of each parasite in their respective hosts, we divided the total number of positive samples by the total sampling population and then multiplied the result by 100. Data were analysed using GraphPad Prism, 2007. The chi‐square tests were used to calculate probability (*p*) values by comparing parasite‐infected faecal samples among different hosts for a particular parasite species. Fisher's exact tests (two‐sided) were used to compare the data between zoonotic and non‐zoonotic parasites. The *p* values less than 0.05 were considered to be statistically significant. Figures were drawn by using OriginPro 2024.

## 3 Results

**FIGURE 3:**
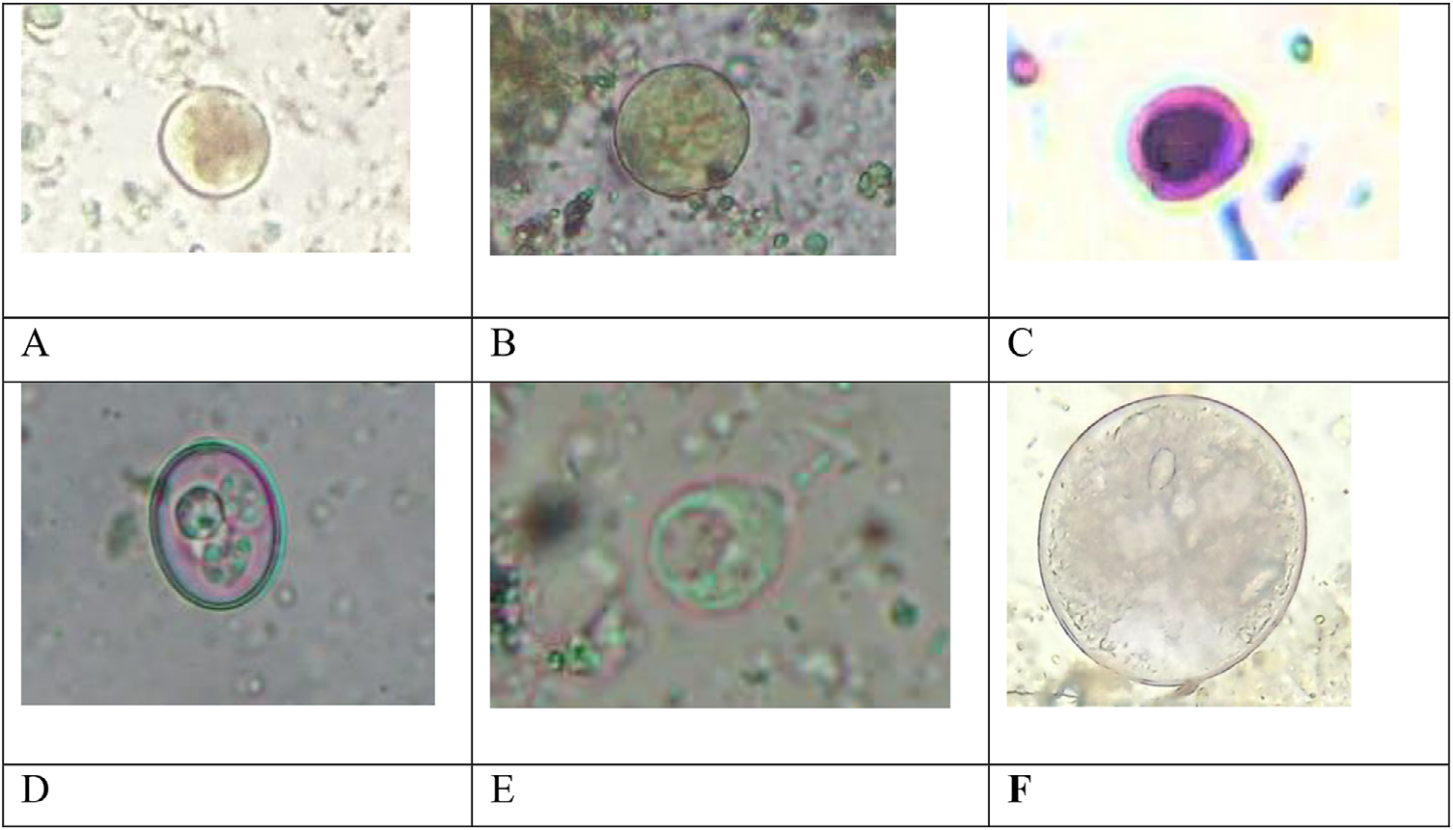
Gastrointestinal protozoan parasites (cysts/oocysts at 40× and 100× magnifications of the objective lens of a microscope). (A) Cyst of *Entamoeba* sp. (buffalo), (B) cyst of *Entamoeba coli* (pig), (C) oocyst of *Cryptosporidium* sp. (buffalo), (D) oocyst of *Eimeria* sp. (buffalo), (E) cyst of *Blastocystis* sp. (pig) and (F) cyst of *Balantidium coli* (pig).

**FIGURE 4:**
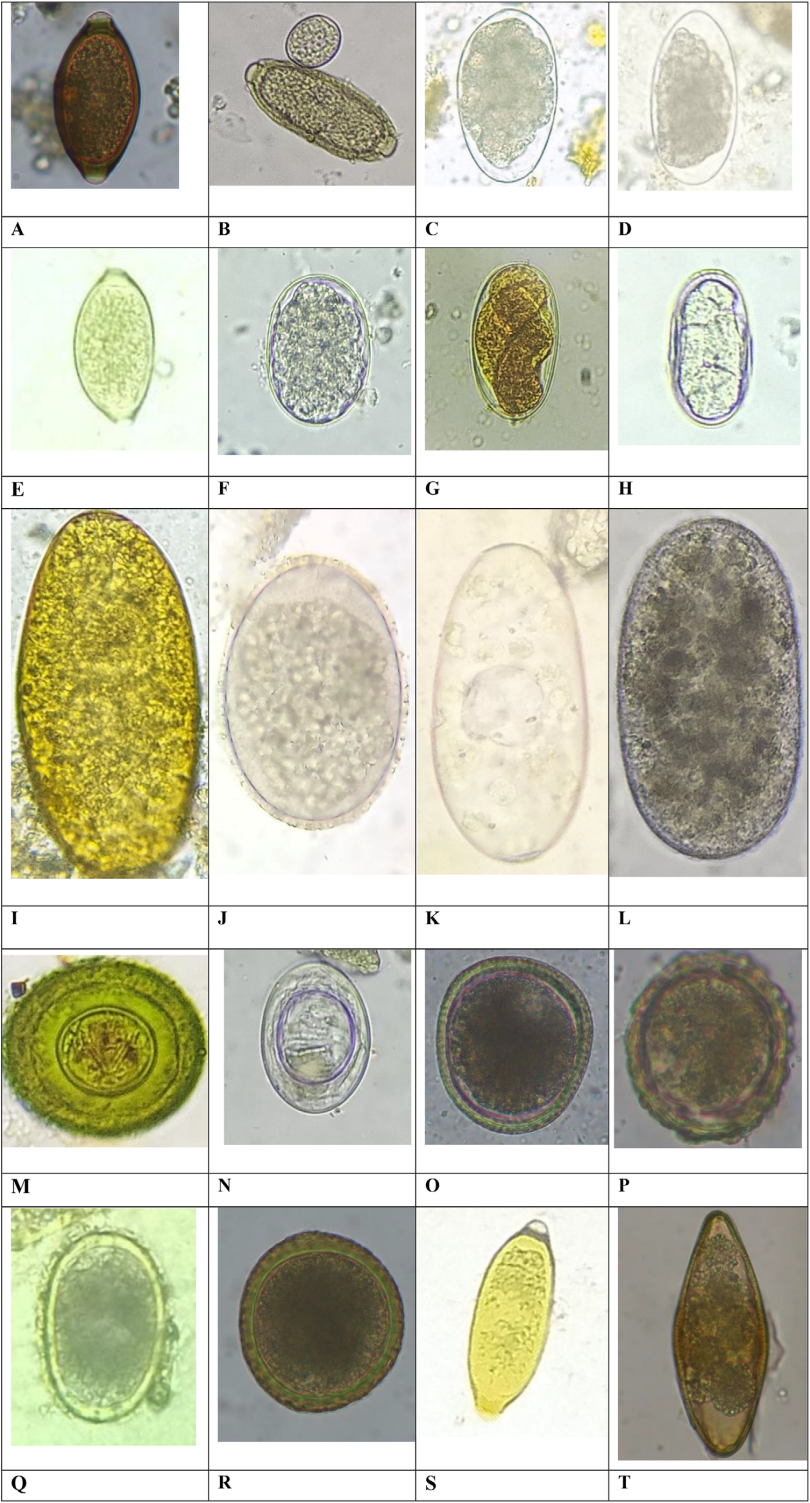
Gastrointestinal helminth parasites (eggs at 40× magnifications of the objective lens of a microscope). (A) Egg of *Trichuris* sp. (dog), (B) egg of *Capillaria* sp. (1) (two polar ends) and oocyst of *Eimeria* sp. (oval small) (rat), (C) egg of strongyle (1) (goat), (D) egg of *Trichostrongylus* sp. (goat), (E) egg of *Capillaria* sp. (2) (rat), (F) egg of hookworm (dog), (G) egg of *Strongyloides* sp. (pig), (H) egg of strongyle (2) (rat), (I) egg of *Fasciola* sp. (cow), (J) egg of strongyle (3) (cow), (K) egg of *Paramphistomum* sp. (buffalo), (L) egg of strongyle (4) (pig), (M) egg of *Hymenolepis diminuta* (rat), (N) egg of *Hymenolepis nana* (rat), (O) egg of *Toxocara canis* (dog), (P) egg of *Ascaris suum* (pig), (Q) egg of *Ascaridia galli* (chicken), (R) egg of *Toxocara vitulorum* (buffalo), (S) egg of *Capillaria* sp. (3) (rat) and (T) egg of *Oxyurid* sp. (rat).

The coproscopy revealed that 100% (117/117) of the sampled animals were infected with at least one species of GIPs. Similarly, 28 different species of GIPs, including single‐celled protozoa and helminths, were reported (Figures 3 and 4; Table S1). Out of the 28 species, helminth parasites (85.5%; 19 species) showed higher prevalence and diversity than protozoa (75.2%; nine species); however, data were not statistically significant (*p* > 0.05). Based on the literature, 21 of them had the potential to be transmitted to humans. This includes protozoa such as *Balantidium coli*, *Blastocystis* sp., *Cryptosporidium* sp., *Entamoeba coli*, *Entamoeba* sp., *Giardia* sp. and *Toxoplasma gondii* and helminths such as *Ascaris suum*, *Capillaria* sp., *Fasciola* sp., *Oxyurid* sp., *Toxocara canis*, *Toxocara cati*, *Toxocara vitulorum*, hookworm, *Hymenolepis nana*, *Hymenolepis diminuta*, *Strongyloides* sp., Taeniid, *Trichostrongylus* sp. and *Trichuris* sp. Overall prevalence rates indicated that *Entamoeba* sp. (47%) was the highest, followed by *Cryptosporidium* sp. (26.5%) in the case of protozoa. In contrast, the rates were highest in hookworm (15.4%), followed by *Trichuris* sp. (13.7%) and *Trichostrongylus* sp. (12.8%) in the context of helminths  (Table S1). The study found the predominance of zoonotic helminths (70.1%) over zoonotic protozoa (47.9%). Statistical analysis using Fisher's exact test indicated significant differences (*p* < 0.05) (Table 1). Furthermore, the overall prevalence rate of zoonotic protozoa was higher than non‐zoonotic protozoa (47.9% vs. 32.5%) (*p *< 0.05). Similarly, zoonotic helminths were significantly more prevalent than non‐zoonotic helminths (70.1% vs. 30.8%) (*p* < 0.05) (Table 1). Notably, pigs were reported to harbour the highest number of zoonotically significant parasites (12 species), followed by cattle and dogs (8 species), while the rest of all other hosts, like buffaloes, cats, goats, chickens and rats, harboured at least six zoonotic species (Table S1). Notably, a few species without any zoonotically significant parasites for human health were also recorded, such as *Cystoisospora* sp., *Eimeria* sp., *Ascaridia galli*, *Spirocera lupi*, strongyle, *Paramphistomum* sp. and *Moniezia* sp. (Table S1).

**TABLE 1:** Prevalence rates (%) of gastrointestinal parasites of zoonotic and non‐zoonotic significance in different hosts.

GI parasites	Cattle (*N*1 = 30)	Buffaloes (*N*2 = 15)	Pigs (*N*3 = 20)	Cats (*N*4 = 5)	Dogs (*N*5 = 15)	Rats (*N*6 = 12)	Chickens (*N*7 = 5)	Goats (*N*8 = 15)	Total (*N*) = 117	*p* values (Fisher's exact test, two‐sided)
Protozoa										
Zoonotic protozoa	12 (40)	5 (33.3)	17 (85)	3 (60)	8 (53.3)	3 (25)	2 (40)	6 (40)	56 (47.9)	< 0.05
Non‐zoonotic protozoa	13 (43.3)	4 (26.7)	6 (30)	1 (20)	4 (26.7)	3 (25)	2 (40)	5 (33.3)	38 (32.5)	
Helminths										
Zoonotic helminths	15 (50)	7 (46.7)	16 (80)	5 (100)	14 (93.3)	12 (100)	3 (60)	10 (66.7)	82 (70.1)	< 0.05
Non‐zoonotic helminths	13 (43.3)	9 (60)	5 (25)	0 (0)	2 (13.3)	3 (25)	1 (20)	3 (20)	36 (30.8)	
Total										
Total protozoa	24 (80)	11 (73.3)	18 (90)	4 (80)	10 (66.7)	6 (50)	3 (60)	12 (80)	88 (75.2)	> 0.05
Total helminths	22 (73.3)	13 (86.7)	18 (90)	5 (100)	15 (100)	12 (100)	4 (80)	11 (73.3)	100 (85.5)	
Grand total	30 (100)	15 (100)	20 (100)	5 (100)	15 (100)	6 (100)	5 (100)	15 (100)	117 (100)	

**FIGURE 5:**
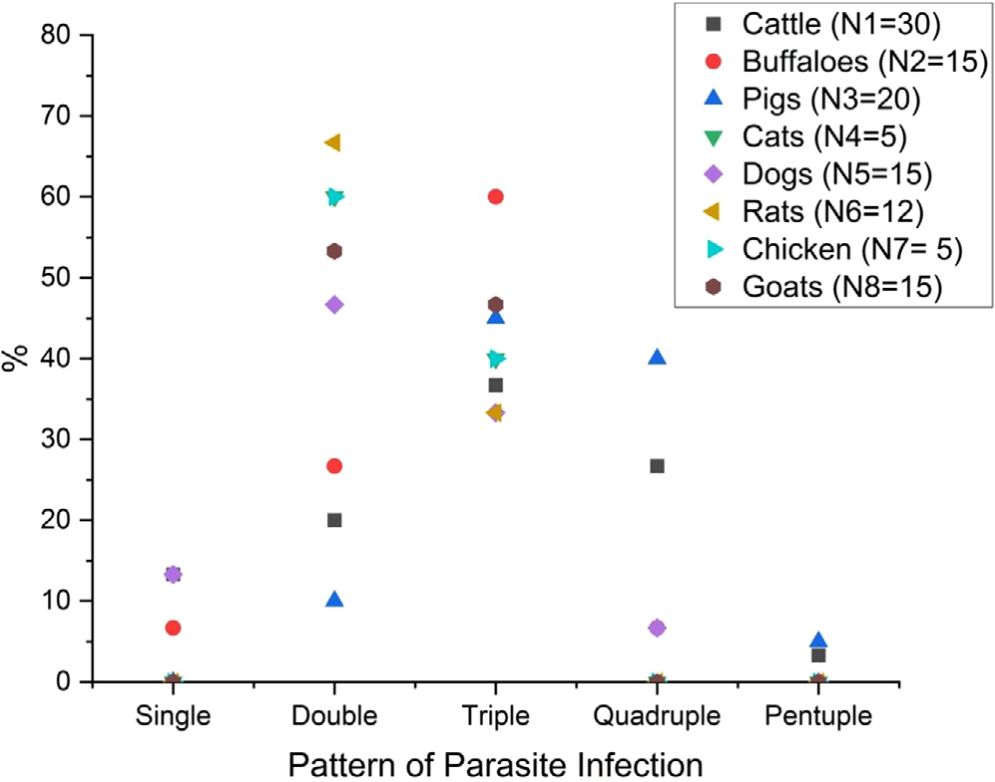
Pattern of parasite infection (drawn by OriginPro 2024).

Furthermore, 94% (110/117) of the sampled animals were found to be excreting eggs/oocysts/cysts of two or more than two parasites, while the rest 6% (7/117) of the animals excreting the eggs/oocysts/cysts of a single species of parasite. This indicates the dominance of polyparasitism. Co‐infection with three species of parasites was the most common (41.9%), followed by two (35%), while the pentuplet pattern of infection was the least common (1.7%) (Figure 5). The pattern of overall parasitic infections among the sampled animals was statistically significant (*p *< 0.05).

Considering the parasitic burden imposed by the GIPs as evaluated by a McMaster technique, the eggs per gram (EPG) of the zoonotic parasites among various sampled hosts follow the following order: Ascarid, hookworm, *Trichuris* sp., *Strongyloides* sp., *Trichostrongylus, Capillaria* sp., *H. nana*, *Oxyruid* sp. and *H. diminuta* (Table 2).

**TABLE 2:** Parasitic burden eggs per gram (EPG) released by various animal hosts.

Parasites	Host	EPG (ranges)
*Toxocara canis*	Dogs	1000–18,500
*Toxocara cati*	Cats	200–16,000
*Toxocara vitulorum*	Cattle/buffaloes	300–13,500
Hookworm	Pigs/cats/dogs	200–12,500
*Ascaris suum*	Pigs	100–10,000
*Trichuris* sp.	Pigs/dogs	200–7,200
*Strongyloides* sp.	Cattle/buffaloes/pigs/goats	100–2,800
*Trichostrongylus*	Goats/chicken	100–1,600
*Capillaria* sp.	Cats/dogs/rats	100–1,200
*Hymenolepis nana*	Rats	100–1,000
*Hymenolepis diminuta*	Rats	100–800

## 4 Discussion

Both protozoa and helminth parasites occurring in animals are well‐known threats to clinical settings because many can potentially cause zoonotic diseases. Here, we reported 21 zoonotic parasites from eight different animal hosts along the Karmanasa River bank in central Nepal.

In the current study, the finding of protozoa with a high prevalence was *Entamoeba* sp. Per our findings, several studies have reported *Entamoeba* infections in livestock such as pigs (R. B. Adhikari, Adhikari Dhakal, et al. 2021), cattle (Jawad, Jawad, and Al‐Fatlawi 2023), buffaloes (R. B. Adhikari, Adhikari Dhakal, and Ghimire 2022) and goats (Ghimire and Bhattarai 2019), as well as pets such as cats (R. B. Adhikari, Adhikari Dhakal, et al. 2023), dogs (R. B. Adhikari, Adhikari Dhakal, and Ghimire 2023) and rodents (Mohebali et al. 2017). Amoebiasis is primarily transmitted through faecal‐oral contact or oral intake of quadrinucleated cysts, making it a challenging threat to human health due to its zoonotic nature and easy transmission route. Although *Entamoeba bovis*, commonly occurring in the GI tract of domestic animals such as buffaloes, cattle and goats, does not infect humans (Aryal et al. 2022), other species, such as *E* *ntamoeba* *histolytica*, *Entamoeba dispar*, *E. coli*, *Entamoeba moshkovskii*, *E* *ntamoeba* *hartmanni* and *Entamoeba polecki* have been identified in clinical settings (Dong et al. 2017; Ngui et al. 2012; Stensvold et al. 2018). Similarly, *Blastocystis* subtypes (ST1, ST3 and ST5) have been detected in porcine hosts (Masuda et al. 2022). Although human infection with these strains is sporadic, reports of possible zoonosis have been identified in Thailand (Ruang‐Areerate et al. 2021) and Australia (Wang et al. 2014), suggesting the risk of cross‐contamination through intimate contact with pigs in the study area. Likewise, *Giardia* sp. has been reported in 8.6% of faeces, and *Giardia lamblia* alone causes giardiasis in humans and most mammals (Feng and Xiao 2011). This indicates that the presence of *Giardia* cyst in the faecal samples of cattle, pigs and goats in the open landscape in study areas potentially adds a significant risk. Furthermore, livestock and free‐ranging canids and felines have been reported to harbour coccidian parasites such as *Cryptosporidium* sp. (26.5%) and *T. gondii* (0.9%). *Cryptosporidium* spp. cause intestinal cryptosporidiosis, a severe illness that potentially infects immunocompromised humans and animals (Pumipuntu and Piratae 2018). Therefore, a chance for cross‐transmission via the faecal–oral route in the study area exists since sporulated oocysts are environmentally resistant and remain viable for more extended periods in the open environment. Comparably, *T. gondii*, whose preferred host is the felid (cat) family, is also known to induce congenital toxoplasmosis and miscarriage in both humans and livestock (Dubey and Beattie 1988). Remarkably, a 53‐day‐old infant died in Nepal after being diagnosed with congenital toxoplasmosis in 2011 (Rai et al. 2011). Hence, it is crucial to understand these zoonotic pathogens in co‐habiting hosts within the human periphery, suggesting a need for molecular study for species identification.

*B. coli* is the only ciliate reported in the current study. It was reported in the faecal samples of cattle (26.7%), buffaloes (26.7%) and pigs (35%). Even if pigs are considered the reservoir hosts (Schuster and Ramirez‐Avila 2008), these ciliates can survive and grow in the external environment at 25–40°C (Clark and Diamond 2002). They can be easily transmitted to humans via the consumption of infective cystic stage.

Furthermore, the finding of helminths with a higher prevalence in the current study was Ascarid spp. These geo‐helminths were also reported in almost every host in the current study. Except for *A. galli*, an intestinal nematode of birds and poultry, all other roundworms, especially *T. cati* and *T. canis* associated with cats and dogs, respectively, are responsible for human toxocariasis (Bhangale, Narladkar, and Tayde 2021). Even if these worms are unable to mature into adults, their larvae wander through the liver, brain and other visceral organs, causing hepatitis, pneumonia, meningoencephalitis, spasmodic pain in the abdomen and eosinophilia, including behavioural and cognitive deformities in humans (Luna et al. 2018). On the other hand, *T. vitulorum* is linked to disease, economic losses, and higher mortality rates in bovine or buffalo calves. Still, it is a minor factor contributing to larva migrans, primarily affecting children in tropical regions (CFSPH 2016). In the same way, *A. suum* is also a well‐known pig‐associated zoonotic parasite (Nejsum et al. 2012). Therefore, the presence of infected pigs and piglets excreting the eggs of these geo‐helminths in the open landscape in the study area might be a risk factor. Furthermore, people using animal dung as manure in their agricultural fields potentially aids in human cross‐transmission.

Another geo‐helminth, hookworm, was reported in about 16% of the faecal samples of animals, like pigs and free‐ranging cats and dogs. In general, dogs and cats can harbour zoonotic hookworm species, such as *Ancylostoma braziliense*, *Ancylostoma caninum*, *Ancylostoma ceylanicum*, and *Uncinaria stenocephala* (CDC 2023), and both pigs and dogs are the proven transport hosts of *Necator americanus*, a human strand of hookworm (Boyko et al. 2020). Notably, animal hookworms typically do not develop in the human intestine. Instead, it infects extra‐intestinal sites such as the skin, resulting in cutaneous larval migrants (CDC 2023), indicating the cross‐transmission risk in the study site. Furthermore, *Trichuris* sp. is another soil‐transmitted helminth reported in 13.7% of faecal samples in the current study. Besides trichuriasis caused by *Trichuris trichiura*, a human strand of whipworm, infection with *Trichuris vulpis* and *Trichuris suis*, associated with canine and porcine hosts, respectively, has also been reported in humans in usual contact with these hosts in rural settings (Areekul et al. 2010; Phosuk et al. 2018). Another zoonotic nematode, *Trichostrongylus* sp., was reported in almost 13% of the faecal samples. It has been reported in cattle, buffaloes, goats, pigs and chickens. Despite human trichostrongylosis being reported sporadically, the infection may occur upon ingesting infective‐stage larvae (Boupha et al. 2011). Similarly, *Capillaria* sp. was reported in 7.7% of the faecal samples, and the eggs were recorded in the faeces of cattle, free‐ranging cats and dogs, rats, and chickens. Capillariasis due to *Capillaria philippinensis*, *Capillaria hepatica*, *Capillaria plica* and *Capillaria aerophila* has been reported in humans (Cross 1992; Pal and Gutama 2024), and the infection follows ingestion of contaminated water, soil, vegetables or larvae‐infested fishes, implying poor hygiene and interaction with rodents or other animals possibly contribute the risk (Pal and Gutama 2024).

Notably, the eggs of *Oxyruid* sp. were reported from the faeces of rats only. In general, rats harbour pinworms such as *Syphacia muris*, *Syphacia obvelata* and *Aspiculuris tetraptera*, of which the zoonotic potential of *Syphacia* spp. has been confirmed (Mahmoud et al. 2009). Similarly, hymenolepiasis caused by tapeworms such as *H. nana* and *H. diminuta*, harboured by these rodents, also possess zoonotic potentialities (King and Fairley 2015; Mercado and Arias 1995), indicating infected rats also point out the infection risk to humans.

Here, we reported that parasitic infection in a single host by multiple species of GIPs was highly dominated over infection by a single species of GIP. In general, concurrent infection is a norm, which may result in enhanced competition among the co‐habiting parasites, thereby reducing the host's fitness (Bordes and Morand 2011). It can also make the host more susceptible to predation or diseases (Irvine et al. 2006). For instance, a laboratory rat infected with *Trypanosoma lewisi* and *T. gondii* had more tachyzoites than those parasitized by *T. gondii* alone (Guerrero, Chinchilla, and Abrahams 1997). Similarly, experimentally infected piglets with both *A. suum* larvae and *Escherichia coli* showed severe respiratory distress and weight loss due to synergistic effects (Adedeji, Ogunba, and Dipeolu 1989). In our previous study, a male buffalo calf heavily infested with multiple protozoa and helminth GIPs had severe GI pathology (R. B. Adhikari and Ghimire 2021). However, negative interaction among co‐habiting GIPs can also occur.

Furthermore, the McMaster technique employed to assess the parasitic burden indicated that the participating animals were excreting moderate to large numbers of zoonotic parasite eggs in each gram of their faeces in the open landscape of the study area. In this scenario, we speculate three possible ways parasitic zoonosis might occur at the study site. First, the inhabitant uses river water contaminated with faeces to wash clothes, irrigate their agricultural lands and feed their livestock. Secondly, seasonal flooding carries animal faeces, other wastes and dirt into human‐inhabitant areas. Finally, there is increasing human‐animal contact due to the domestication of animals for food items, such as milk, meat and companionship. It also involves using the typical landscape for multiple purposes, such as pasturelands for animal grazing by farmers, playgrounds for children and recreational spots for older adults and visitors. With their increasing exposure to the open landscape along the Karmanasa River bank, local inhabitants, recreational visitors, and school children are at greater risk of acquiring parasitic diseases. However, further epidemiological studies are needed to verify these notions.

## 5 Conclusion

The study along the Karmanasa River bank revealed that the faecal samples of openly defaecating livestock, free‐ranging canids and felines and rodents harboured greater diversity and a significantly higher prevalence rate of zoonotically essential parasites. Besides, it remains the first study to describe such a wide variety of GIPs from the rat population in Nepal. Among the 28 GIPs detected, 21 have the potential to infect humans, signifying that co‐habiting humans, either local inhabitants or recreational visitors, including children, have a greater risk of acquiring zoonotic intestinal parasitic diseases. Therefore, one health concept of research, which involves assessing the presence of intestinal parasites in environmental factors, like river water and soil, animals and humans in the study area, should be conducted shortly. A study of this kind will undoubtedly increase our understanding of parasitic zoonosis among humans, animals and the environment and contribute to maintaining a healthy coexistence of humans, livestock and free‐ranging animals in the study area.

## Author Contributions

**Roshan Babu Adhikari**: conceptualization, methodology, field and laboratory investigation, writing–original draft, formal analysis, data curation, software, writing–review and editing. **Diksha Ghimire**: conceptualization, field survey, data curation, writing–review and editing. **Tirth Raj Ghimire**: conceptualization, data analysis, software, writing–review and editing.

## Ethics Statement

The authors declare that the study was conducted on naturally infected animals. No experimental infection was established during this research work. The required permission for collecting the faecal samples was issued by Lalitpur Metropolitan Municipality, Lalitpur (Permission No. 2950/077/078), Mahalaxmi Municipality, Lalitpur (Permission No. 3071/080/081) and Alka Health Institute Pvt. Ltd., Lalitpur (Permission No.25/079/080).

## Conflicts of Interest

The authors declare no conflicts of interest.

### Peer Review

The peer review history for this article is available at https://publons.com/publon/10.1002/vms3.70164.

## Supporting information



Supporting information

## Data Availability

All data generated in the research have been submitted in this article.
