# Supplementary material for: Investigation of the Occurrence of Zoonotic Intestinal Parasites along the Karmanasa River Bank in Lalitpur, Nepal
Source: Vet Med Sci. 2024 Dec 18;11(1):e70164. doi: 10.1002/vms3.70164 (PMC11653088; doi:10.1002/vms3.70164)
Supplement: Supplementary file 1 — Supporting information [file VMS3-11-e70164-s001.docx]

| **GI Parasites** | **Cattle (N1=30)** | **Buffaloes**  **(N2=15)** | **Pigs**  **(N3=20)** | **Cats (N4=5)** | **Dogs (N5= 15)** | **Rats (N6= 12)** | **Chickens (N7= 5)** | **Goats (N8= 15)** | **Total (N=117)** | **p-values (Chi-square tests)** |
| --- | --- | --- | --- | --- | --- | --- | --- | --- | --- | --- |
| **Protozoa** | | | | | | | | | |  |
| ***Entamoeba* sp.*** | 13 (43.3) | 9 (60) | 15 (75) | 1 (20) | 4 (26.7) | 3 (25) | 1 (20) | 9 (60) | 55 (47) | <0.05 |
| ***Entamoeba coli**** | 0 (0.0) | 0 (0.0) | 1 (5) | 0 (0.0) | 0 (0.0) | 0 (0.0) | 0 (0.0) | 0 (0.0) | 1 (0.9) | ns |
| ***Giardia* sp. *** | 2 (6.7) | 0 (0.0) | 6 (30) | 0 (0.0) | 0 (0.0) | 0 (0.0) | 0 (0.0) | 2 (13.3) | 10 (8.5) | <0.05 |
| ***Cryptosporidium* sp. *** | 10 (33.3) | 3 (20) | 5 (25) | 3 (60) | 4 (26.7) | 0 (0.0) | 2 (40) | 4 (26.7) | 31 (26.5) | ns |
| ***Balantidium coli**** | 8 (26.7) | 4 (26.7) | 7 (35) | 0 (0.0) | 0 (0.0) | 0 (0.0) | 0 (0.0) | 0 (0.0) | 19 (16.2) | <0.05 |
| ***Blastocystis* sp. *** | 0 (0.0) | 0 (0.0) | 1 (5) | 0 (0.0) | 0 (0.0) | 0 (0.0) | 0 (0.0) | 0 (0.0) | 1 (0.9) | ns |
| ***Cystoisospora* sp.** | 0 (0.0) | 0 (0.0) | 3 (15) | 1 (20) | 4 (26.7) | 0 (0.0) | 0 (0.0) | 0 (0.0) | 8 (6.8) | <0.05 |
| ***Eimeria* sp.** | 13 (43.3) | 4 (26.7) | 3 (15) | 0 (0.0) | 0 (0.0) | 3 (25) | 2 (40) | 5 (33.3) | 30 (25.7) | ns |
| ***Toxoplasma gondii**** | 0 (0.0) | 0 (0.0) | 0 (0.0) | 1 (20) | 0 (0.0) | 0 (0.0) | 0 (0.0) | 0 (0.0) | 1 (0.9) | <0.05 |
| **Helminths** | | | | | | | | | |  |
| ***Toxocara vitulorum**** | 4 (13.3) | 3 (20) | 0 (0.0) | 0 (0.0) | 0 (0.0) | 0 (0.0) | 0 (0.0) | 0 (0.0) | 7 (6) | ns |
| ***Ascaris suum* *** | 0 (0.0) | 0 (0.0) | 4 (20) | 0 (0.0) | 0 (0.0) | 0 (0.0) | 0 (0.0) | 0 (0.0) | 4 (3.4) | <0.05 |
| ***Ascaridia galli*** | 0 (0.0) | 0 (0.0) | 0 (0.0) | 0 (0.0) | 0 (0.0) | 0 (0.0) | 2 (40) | 0 (0.0) | 2 (1.7) | <0.05 |
| ***Toxocara canis**** | 0 (0.0) | 0 (0.0) | 0 (0.0) | 0 (0.0) | 4 (26.7) | 0 (0.0) | 0 (0.0) | 0 (0.0) | 4 (3.4) | <0.05 |
| ***Toxocara cati**** | 0 (0.0) | 0 (0.0) | 0 (0.0) | 2 (40) | 0 (0.0) | 0 (0.0) | 0 (0.0) | 0 (0.0) | 2 (1.7) | <0.05 |
| ***Trichuris* sp. *** | 0 (0.0) | 0 (0.0) | 6 (30) | 0 (0.0) | 3 (20) | 2 (16.7) | 2 (40) | 3 (20) | 16 (13.7) | <0.05 |
| ***Capillaria* sp. *** | 1 (3.3) | 0 (0.0) | 0 (0.0) | 1 (20) | 1 (6.7) | 5 (41.7) | 1 (20) | 0 (0.0) | 9 (7.7) | <0.05 |
| ***Hymenolepis nana**** | 0 (0.0) | 0 (0.0) | 0 (0.0) | 0 (0.0) | 0 (0.0) | 4 (33.3) | 0 (0.0) | 0 (0.0) | 4 (3.4) | <0.05 |
| ***Hymenolepis diminuta**** | 0 (0.0) | 0 (0.0) | 0 (0.0) | 0 (0.0) | 0 (0.0) | 5 (41.7) | 0 (0.0) | 0 (0.0) | 5 (4.3) | <0.05 |
| **Hookworm*** | 0 (0.0) | 0 (0.0) | 7 (35) | 3 (60) | 8 (53.3) | 0 (0.0) | 0 (0.0) | 0 (0.0) | 18 (15.4) | <0.05 |
| ***Strongyloides* sp*.* *** | 2 (6.7) | 1 (6.7) | 3 (15) | 0 (0.0) | 2 (13.3) | 0 (0.0) | 1 (20) | 2 (13.3) | 11 (9.4) | ns |
| **Strongyle** | 12 (40) | 5 (33.3) | 4 (20) | 0 (0.0) | 0 (0.0) | 3 (25) | 0 (0.0) | 4 (26.7) | 28 (23.9) | ns |
| ***Trichostrongylus* sp*.**** | 4 (13.3) | 3 (20) | 3 (15) | 0 (0.0) | 0 (0.0) | 0 (0.0) | 1 (20) | 4 (26.7) | 15 (12.8) | ns |
| **Taeniid*** | 0 (0.0) | 0 (0.0) | 0 (0.0) | 0 (0.0) | 3 (20) | 0 (0.0) | 0 (0.0) | 0 (0.0) | 3 (2.6) | <0.05 |
| ***Spirocera lupi*** | 0 (0.0) | 0 (0.0) | 0 (0.0) | 0 (0.0) | 2 (13.3) | 0 (0.0) | 0 (0.0) | 0 (0.0) | 2 (1.7) | ns |
| **Oxyurid sp. *** | 0 (0.0) | 0 (0.0) | 0 (0.0) | 0 (0.0) | 0 (0.0) | 3 (25) | 0 (0.0) | 0 (0.0) | 3 (2.6) | <0.05 |
| ***Fasciola* sp. *** | 6 (20) | 3 (20) | 1 (5) | 0 (0.0) | 0 (0.0) | 0 (0.0) | 0 (0.0) | 1 (6.7) | 11 (9.4) | ns |
| ***Paramphistomum* sp.** | 7 (23.3) | 4 (26.7) | 0 (0.0) | 0 (0.0) | 0 (0.0) | 0 (0.0) | 0 (0.0) | 0 (0.0) | 11 (9.4) | <0.05 |
| ***Moniezia* sp.** | 1 (3.3) | 1 (6.7) | 0 (0.0) | 0 (0.0) | 0 (0.0) | 0 (0.0) | 0 (0.0) | 3 (20) | 5 (4.3) | ns |

**Supplementary 1.** Prevalence (%) of gastrointestinal parasites of sampled animals (* indicates zoonotic species)
